# Norovirus Attachment and Entry

**DOI:** 10.3390/v11060495

**Published:** 2019-05-30

**Authors:** Vincent R. Graziano, Jin Wei, Craig B. Wilen

**Affiliations:** Departments of Laboratory Medicine and Immunobiology, Yale University School of Medicine, New Haven, CT 06520, USA; vincent.graziano@yale.edu (V.R.G.); Jin.wei@yale.edu (J.W.)

**Keywords:** norovirus entry, CD300lf, JAM-A, bile salts, histo-blood group antigens, murine norovirus, viral tropism

## Abstract

Human norovirus is a major human pathogen causing the majority of cases of viral gastroenteritis globally. Viral entry is the first step of the viral life cycle and is a significant determinant of cell tropism, host range, immune interactions, and pathogenesis. Bile salts and histo-blood group antigens are key mediators of norovirus entry; however, the molecular mechanisms by which these molecules promote infection and the identity of a potential human norovirus receptor remain unknown. Recently, there have been several important advances in norovirus entry biology including the identification of CD300lf as the receptor for murine norovirus and of the role of the minor capsid protein VP2 in viral genome release. Here, we will review the current understanding about norovirus attachment and entry and highlight important future directions.

## 1. Background

Human norovirus (HNoV) is the leading cause of acute gastroenteritis worldwide causing roughly 700 million infections and 200,000 deaths annually [1,2]. Currently, there are no licensed antiviral treatments or vaccines due in part to our incomplete understanding of HNoV biology [3,4]. The host genes required for HNoV infection, in vivo cell tropism, and mechanisms of immunity and immune evasion remain unclear. Viral entry is the first stage of the virus life cycle. It is initiated by virus binding to the cell surface and culminates in the release of the viral genome into the host cell cytoplasm [5]. Elucidating the molecular mechanisms by which a virus enters its target cell has important implications for host range, cell tropism, and pathogenesis [5,6]. In addition, viral entry represents a well-validated therapeutic target for both small-molecule antagonists and vaccines that elicit neutralizing antibodies [7]. Here, we will review the current understanding of norovirus entry, highlight key recent advances, and propose potential strategies to further understand norovirus entry and pathogenesis.

The *Caliciviridae* family is a group of non-enveloped, positive-sense single-stranded RNA (ssRNA) viruses that includes five genera, of which *Norovirus* and *Sapovirus* genera are significant human pathogens [8,9,10,11]. The *Norovirus* genus is subdivided into seven genogroups. Genogroup (G) I viruses infect humans, GII viruses infect humans and pigs, GIII viruses infect cows and sheep, GIV viruses infect humans, cats, and dogs, GV viruses infect mice, GVI viruses infect dogs and cats, and GVII viruses infect dogs [12,13,14,15,16,17,18,19]. The norovirus genome is 7.5 kilobases in length and encodes six non-structural proteins and two structural proteins, i.e., the major capsid protein VP1 (59 kDa) and the minor capsid protein VP2 (29 kDa) [3]. Norovirus particles are approximately 27–30 nm in diameter with T = 3 icosahedral symmetry, although smaller virions with T = 1 icosahedral symmetry have been described [20]. The viral capsid is comprised of 90 dimers of VP1, each of which is comprised of a shell (S) domain and a protruding (P) domain which are connected by a flexible linker [21]. The S domain is the mostly highly conserved VP1 domain and is sufficient for the assembly of the norovirus capsid shell, which encapsulates the viral genome [21]. In contrast, the P domain is more variable and includes a P1 and a P2 subdomain that are discontinuous in primary amino acid sequence. The highly variable P2 subdomain represents the outermost structure of VP1 and is made up of six anti-parallel β-barrels extended out from P1. The P1 domain, which consists of one α helix and eight β sheets links, the S and P2 domains. The P2 subdomain is a target for neutralizing antibodies and contains the defined and putative receptor binding site for mouse norovirus (MNoV) and HNoV, respectively [22,23,24,25,26,27].

Studies on HNoV entry have been hindered by the lack of infectious molecular clones and challenges with in vitro HNoV culture. Much of our current understanding about norovirus entry comes from biochemical studies with HNoV-like particles and the related viruses MNoV and feline calicivirus (FCV), as there exists infectious molecular clones and highly susceptible cell lines for these viruses [3,24,28,29,30]. However, significant recent advances in HNoV culture systems in both human intestinal enteroids and lymphoid cell lines are poised to accelerate our understanding of HNoV entry [31,32]. Additionally, recent genome-wide CRISPR screens identified CD300lf as the proteinaceous receptor for MNoV, the first receptor described of any norovirus [26,33], while recent cryo-electron microscopy studies revealed insight into the molecular mechanism of FCV entry and viral genome delivery [34,35,36]. These studies have important implications in understanding HNoV entry and pathogenesis.

## 2. The Role of Glycans in Norovirus Attachment

**Figure 1:**
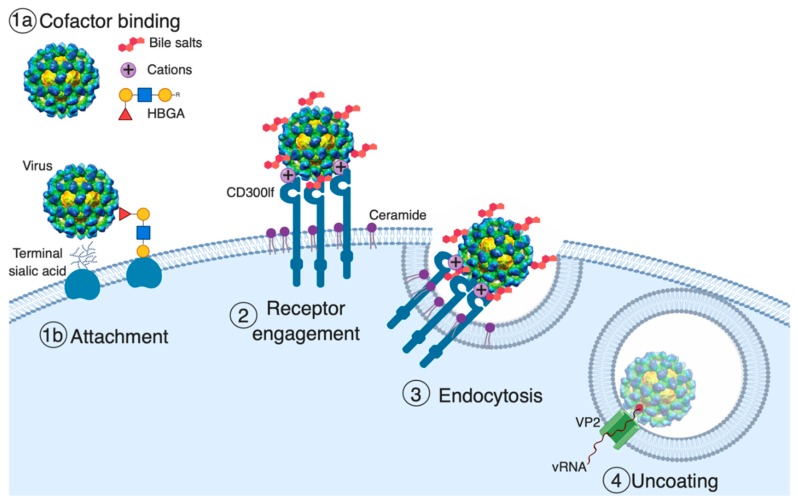
Model of norovirus entry. The first and often rate-limiting step of viral entry is viral attachment to the cell surface. Cell-associated host glycans including terminal sialic acid and histo-blood group antigens (HBGAs) can facilitate the entry of mouse (MNoV) and human norovirus (HNoV), respectively [37,39,40,41]. Soluble cofactors including soluble forms of HBGAs (HNoV), bile salts (MNoV and HNoV), and divalent cations (MNoV) can also augment the attachment of the virus to cells [25,26,31,38,41]. For MNoV, these soluble cofactors increase virus attachment in a receptor-dependent manner. The second stage of viral entry is receptor engagement. CD300lf, an immunoglobulin (Ig) domain-containing membrane protein, is the MNoV receptor, and feline junctional adhesion molecule A (fJAM-A) is the feline calicivirus (FCV) receptor, while the HNoV receptor remains unknown [26,33,42]. Interestingly, ceramide alters CD300lf conformation or clustering, promoting the interaction with MNoV. Following receptor engagement, the virus is endocytosed where, at least for FCV, receptor binding triggers the minor capsid protein VP2 to form a membrane portal that may enable viral genome release in the cytosol [34].

Non-enveloped virus entry is a multi-step process starting with viral attachment to target cells, followed by receptor engagement, endocytosis, cell membrane penetration, and uncoating that culminates in the delivery of the viral genome into the cytoplasm [5] (Figure 1). Notably, viral entry is a critical determinant of cell tropism, host range, and pathogenesis. The first and often rate-limiting step of viral entry is virus binding to host cells, which is mediated by both host attachment factors and receptors. Attachment factors are host molecules that concentrate the virus on the cell surface but do not actively induce viral entry [5]. While attachment factors increase the efficiency of viral infection, they are by definition not essential. In contrast, viral receptors are essential host molecules that specifically bind the virus particle, induce a conformational change in the virus, and actively promote viral entry [5]. Notably, in some cases, the classification of host molecules involved in viral entry can be dependent on viral strain, host cell type, and culture conditions. Interestingly for noroviruses, both cell-associated and non-cell-associated host molecules that augment binding, albeit by diverse mechanisms, have been described. These molecules include the attachment factors histo-blood group antigens (HBGAs), bile acids, sialic acid, and divalent cations (Figure 1) [25,26,27,31,32,37,38].

The first experimental HNoV challenge studies were performed in human volunteers in the 1970s with what was then called “Norwalk agent”, as it was originally isolated from students at Bronson Elementary School in Norwalk, Ohio [14,43]. These and subsequent challenge studies revealed individuals with both short-term (<6 months) and long-term immunity to HNoV [14,44,45]. This long-term resistance was later correlated with host secretor status, i.e., the ability to secrete HBGAs into body fluids [39]. The secretor status is determined by mutations in the *FUT2* gene which encodes the enzyme FUT2 (α(1,2) fucosyltransferase) [46,47,48]. FUT2 converts the carbohydrate H antigen type-1 precursor to mature H antigen type-1, which can then be further modified by downstream enzymes into diverse carbohydrate antigens. Approximately, 20–30% of people have polymorphisms in *FUT2* resulting in a non-functional enzyme [39]. These individuals, termed non-secretors, are unable to secrete ABO blood group antigens into their body fluids. Importantly, the presence of anti-HNoV antibodies that block in vitro binding of HBGAs to VP1 correlates with protection to certain HNoV genotypes in humans [49,50,51]. Non-secretors exhibit significant resistance to HNoV genogroups GI.1 and GII.4; however, resistance to HNoV is not absolute, as non-secretors can be infected both experimentally and naturally with some HNoVs [39,52,53,54,55,56]. 

Importantly, HBGAs can be both cell-associated and non-cell-associated, and both forms may play a role in norovirus entry [41]. GI.1 virus-like particles (VLPs) can bind cells from secretor-positive but not secretor-negative individuals [41]. HBGAs directly interact with HNoV, although the implications of this interaction remain unclear. HBGAs bind the P2 domain of VP1, albeit at relatively low affinity (~400 μM) and without inducing significant structural rearrangements in VP1 [57]. Structural and functional studies have described several distinct HBGA binding pockets amongst HNoV strains, which may reflect the ability of given strains to infect non-secretors [58,59,60]. 

While HBGAs represent an important attachment factor for many HNoV strains, further studies are warranted to implicate these glycans as bona fide HNoV receptors. Specifically, a major restriction to HNoV replication in cell lines is at the level of viral entry. Bypassing viral entry by transfection of viral RNA into the cytoplasm of cells is sufficient to produce infectious virions [61]. Of note, one or more post-entry restrictions to HNoV replication are also likely. Importantly, HBGAs are not sufficient to confer susceptibility to infection in these or other human cell lines nor do they explain the limited host range exhibited by HNoVs, as other species that have similar HBGAs are not susceptible to HNoV [61,62]. Further studies are needed to determine the specific role and molecular mechanism of HBGAs in HNoV entry as classical attachment factors or as potential components of a HNoV receptor or coreceptor.

HBGAs are found both in the saliva and associated with cells in the gastrointestinal tract, providing the opportunity for interactions with HNoV. Recent in vitro studies of HNoV have demonstrated that both soluble and cell-associated HBGAs promote cellular infection. Both cell-free and HBGA-expressing bacteria enhance HNoV replication in lymphoid cell lines in vitro [32]. In addition, human intestinal enteroids from secretor-negative individuals were resistant to GII.4 but not GII.3 HNoV strains, consistent with the epidemiology of these genotypes in the human population. Interestingly, exogenous HBGAs are not necessary for successful HNoV replication in the enteroid culture system [31]. The variable susceptibility of non-secretors to HNoV may be due to differences in viral genetics, host genetics, host immunity, and/or environmental factors such as the microbiome [32,52,63].

HBGAs have also been implicated as attachment factors for GIV and GVI canine noroviruses, but they do not appear to play a role in MNoV infection [13,26]. However, other host glycans including sialic acid and heparan sulfate proteoglycans may play a role in viral attachment. Terminal sialic acid (both α2,3- and α2,6-linked) moieties on gangliosides facilitate the attachment of MNoV to cells, while α2,6-linked sialic acid enhanced binding of FCV to target cells [37,64]. The suggested sialic acid binding site on MNoV VP1 closely resembles the HBGA binding site, suggesting that multiple glycans can similarly promote the attachment of diverse noroviruses [65]. Notably, consistent with its role as an attachment factor, sialic acid is not essential for MNoV infection and does not explain the cell tropism or species restriction of MNoV, given the broad expression of sialic acid on diverse cell types from diverse species [26]. 

## 3. The Role of Non-Glycans in Norovirus Attachment

**Figure 2:**
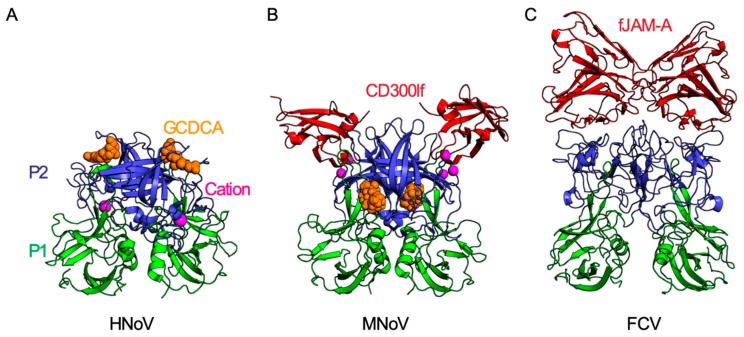
Molecular interactions of calicivirus capsid proteins. Each calicivirus has T = 3 icosahedral symmetry and is comprised of 90 VP1 dimers. Each VP1 capsid monomer is comprised of a shell (S) and a protruding (P) domain. The P domain is further subdivided into an apical P2 domain (blue) and a P1 domain (green) which connects the more variable P2 to the shell domain. (**A**) The secondary bile salt glycochenodeoxycholic acid (GCDCA; shown as orange space-filling model) binds to the apical region of the P2 domain of human norovirus (HNoV) GII.10 (PDB ID: 6GW1) [38]. This binding site partially overlaps with the HBGA binding site on HNoVs (not shown). (**B**) MNoV P2 binds the CD300lf receptor (red) at 1:1 stoichiometry (PDB IB: 6E47). Both cations (magenta) and GCDCA (orange) enhance binding of MNoV to CD300lf. GCDCA binds MNoV VP1 at the dimer interface between the protruding domains, a site different from the GCDCA-binding site on HNoV GII.10 [25,26,27]. (**C**) The apical region of FCV capsid P2 domain binds the FCV receptor fJAM-A (PDB ID: 6GSI). Similar to CD300lf, fJAM-A is an Ig domain-containing membrane protein and binds the P2 domain at 1:1 stoichiometry. While the CD300lf ectodomain is comprised of a single Ig domain, fJAM-A has two Ig domains (D1 and D2) with only the distal D1 domain directly binding the FCV P2 domain [34].

Important host and microbial molecules other than glycans have been reported to also facilitate norovirus attachment. Recently, bile salts were discovered as important cofactors for norovirus infection in vitro [31,38]. Interestingly, bile salts had been previously implicated as important factors for in vitro replication of porcine sapovirus, another member of the *Caliciviridae* family [66]. Bile salts are cholesterol derivatives produced and secreted from the liver into the gastrointestinal tract where they are further modified by intestinal bacteria. Bile salts have diverse physical properties and physiological functions that include facilitating lipid absorption, regulating cellular metabolism, and maintaining intestinal homeostasis [67,68,69]. Recently, Estes and colleagues developed a HNoV culture system in human intestinal enteroids [31]. In this system, exogenous bile was required for replication of HNoV GII.3 isolates and augmented replication of HNoV GII.4 isolates [31]. Notably, bile was required either during or after, but not before, viral adsorption [31]. It was later demonstrated that bile salts can similarly enhance MNoV infection in vitro [25]. The secondary bile salts glycochenodeoxycholic acid (GCDCA) and lithocholic acid (LCA), but not several other bile salts, can bind to a hydrophobic pocket at the dimer interface between the MNoV P1 and P2 subdomains at 1:1 stoichiometry, with a K_D_ of approximately 5 µM [25,38] (Figure 2B). Binding of these secondary bile acids to MNoV VP1 enhanced the ability of the virus to bind cells in a CD300lf receptor-dependent manner. Notably, bile was sufficient but not necessary to increase MNoV binding and infectivity, as other unidentified non-proteinaceous small molecules found in serum could rescue virus attachment [25,26]. Surprisingly, the bile salt binding site on MNoV VP1 did not induce significant structural rearrangements in the P domain and was distant from the CD300lf receptor binding site [25]. More recently, secondary bile salts including GCDCA were shown to also bind the capsid of GII.1, GII.10, and GII.19 HNoV VLPs but not of GI.1, GII.3, GII.4, and GII.17 VLPs [38]. Notably, strain-specific variation was observed in the ability of bile salts to promote HNoV infection of enteroids [31]. Similar to MNoV, bile salts bound to HNoV with 1:1 stoichiometry and low micromolar affinity and induced only modest structural changes in the P domain. However, in contrast to MNoV, the bile salt binding pocket for HNoV was on the apical side of P2, overlapping with the HBGA binding site [38]. The observation that similar bile salts bind both MNoV and HNoV capsid protein suggests a common, yet undetermined, mechanism of action. Whether bile salts induce conformational changes in the native virion that increase cell attachment remains unknown. It is also unclear whether bile salts affect virus stability or contribute to post-binding processes such as uncoating or endosome escape [70]. The role of intestinal bacteria, which metabolize primary bile salts into secondary bile salts, and the effect of bile salts on viral tropism and pathogenesis in vivo also remain unexplored. This may be of particular interest given that commensal bacteria in the intestines are important mediators of MNoV infection [32,63].

In addition to the role of glycans and bile salts, phospholipids were also recently identified as key mediators in norovirus entry. A genome-wide CRISPR screen identified *Sptlcl1* and *Sptlc2* as proviral genes in MNoV infection [26,71]. These genes encode essential proteins for the serine palmitoyltransferase (SPT) complex that catalyzes the rate-limiting biosynthetic step of ceramide and sphingolipids, which are important regulators of membrane fluidity and dynamics [26,72,73]. Interestingly, ceramide has been described as a putative ligand for CD300lf [74]. Sptlc1 and Sptlc2 though important, are not essential, for MNoV binding and infection [26,71]. This binding defect can be rescued by the addition of exogenous ceramide. Ceramide and the SPT complex do not regulate CD300lf expression or membrane trafficking. Rather, ceramide differentially modifies distinct CD300lf antibody epitopes, suggesting that it alters the conformation and/or clustering of CD300lf on the cell membrane to promote viral entry [71]. Ceramide has also been implicated in porcine enteric calicivirus entry, albeit through a different mechanism [75]. Further studies on the role of ceramide in HNoV entry may be valuable in understanding HNoV-receptor interactions.

In summary, both soluble and cell-associated molecules including glycans, bile salts, cations, and phospholipids can augment the binding of noroviruses to host cells. The mechanism of MNoV attachment enhancement can be either receptor-dependent such as for ceramide, cations, and bile salts for MNoV, or receptor-independent such as for HBGAs and sialic acid. These pro-attachment molecules can bind directly to the norovirus capsid or affect the function or conformation of the host cell membrane or receptor. Outstanding questions on norovirus attachment include whether diverse attachment factors are synergistic, how attachment factors promote the binding of the virus at the molecular level, and how attachment factors regulate species, tissue, and cell tropism. Future work is needed to determine the role of such attachment factors in mediating host permissivity to norovirus infection and disease.

## 4. Receptor Engagement

The second stage of viral entry, and the first essential step, is receptor engagement [5]. Host cell receptor utilization informs our understanding of viral pathogenesis, cell and tissue tropism, species tropism, and immune evasion from the humoral immune response. While the HNoV receptor(s) are unknown, recent work with other caliciviruses including FCV and MNoV has informed our understanding about norovirus receptor biology [26,33,34,42,76,77].

Feline Junctional Adhesion Molecule A (fJAM-A) was identified as the receptor for FCV, the first receptor for any member of the *Caliciviridae* family [42]. Interestingly, human JAM-A was recently described as a receptor for San Miguel sea lion virus on human cells [78]. JAM-A is a type I integral membrane protein with two extracellular immunoglobulin (Ig) domains (D1 and D2) [35]. JAM-A localizes to cellular tight junctions and regulates tight junction permeability [79]. fJAM-A expression was both necessary for FCV infection of feline cells and sufficient to enable FCV infection of human cells [42]. The P2 domain of FCV VP1 directly binds the membrane-proximal D1 domain of fJAM-A, which induces a conformational change in the FCV capsid that is required for subsequent viral genome escape [35,36,77] (Figure 2C).

More recently, genome-wide CRISPR screens identified CD300lf as the receptor for MNoV [26,33]. CD300lf is a type I integral membrane protein with a single extracellular Ig-like domain. CD300lf is part of a larger family of CD300 molecules that function as cell death sensors, as they recognize phospholipids typically found on the inner leaflet of cells [80]. Upon binding of these ligands, such as phosphatidylserine and ceramide, CD300lf induces immunoregulatory signals with diverse downstream properties [80]. CD300lf expression is both necessary and sufficient for MNoV infection, as MNoV infection can be inhibited by an anti-CD300lf antibody or genetic disruption of CD300lf [81]. In addition, ectopic expression of murine CD300lf on human and other mammalian cell lines is sufficient to confer cross-species permissivity [26,33]. This further demonstrates that these human cell lines contain the necessary intracellular machinery to efficiently replicate noroviruses, despite their resistance to HNoV [61,62]. Further, CD300lf-deficient mice are resistant to infection with the prototypic MNoV clones CW3 (derived from MNV-1) and CR6 [6,26]. Interestingly, overexpression of a related member of the CD300 family, CD300ld, in non-permissive cells is sufficient for MNoV infection. However, whether physiologic expression of CD300ld contributes to MNoV infection in vitro or in vivo still remains unclear [26,33].

Several mutagenesis and structural studies have determined the molecular mechanisms of CD300lf interactions with MNoV [25,26,27]. On the host side, the ectodomain of CD300lf contains a phospholipid ligand binding pocket that is flanked by two loop regions (CC’ and CDR3) [26]. At the base of this binding pocket is an aspartic acid that coordinates a cation and facilitates ligand binding [82,83]. Interestingly, the CC’ and CDR3 loops are critical for MNoV binding, while the buried aspartic acid is dispensable for infection [26] (Figure 2B). This suggests that MNoV partially mimics phospholipids to productively engage CD300lf [25,26]. On the viral side, the CD300lf ectodomain binds the apical surface of the P2 domain. Notably, this binding pocket on P2 overlaps with the epitopes of previously described neutralizing antibodies and partially overlaps with the corresponding HBGA and bile acid binding sites on HNoV [23,38,58,84,85,86]. The affinity of CD300lf to MNoV is quite low relative to other characterized virus–receptor interactions. Binding studies with soluble CD300lf and MNoV P domains reported a K_D_ of ~25 μm [25]. This suggests that avidity plays a significant role in CD300lf-mediated entry and that MNoV likely engages multiple CD300lf molecules per cell [25].

The discovery of CD300lf as the receptor for MNoV has broadly enhanced our understanding of norovirus entry, tropism, and pathogenesis. First, CD300lf has revealed the mechanism of the species barrier in MNoV infection [26,33]. Second, it has led to increased understanding of the role of bile salts, ceramide, and cations in virus–cell interactions. Third, it has contributed to our understanding of MNoV cell tropism and led to the observation that rare intestinal epithelial cells called tuft cells are infected by enteric MNoV [6]. However, it remains unknown whether HNoV uses a related proteinaceous receptor and whether HBGAs are linked to the HNoV receptor.

## 5. Endocytosis and Uncoating

Upon binding to the cell surface, caliciviruses undergo endocytosis. The viral genome must then escape the capsid and penetrate the endosomal membrane to enter the host cell cytoplasm. Viruses can hijack a number of distinct endocytic routes to gain entry into cells, including micropinocytosis, clathrin-dependent, caveolin-dependent, dynamin-dependent, and cholesterol-dependent pathways [5]. Upon virus internalization, the endosomal location of viral genome release can vary from the early endosome to the endoplasmic reticulum [5]. The various endosomal compartments have different pHs, and pH-dependence has been reported to be essential for other viruses [7,87,88].

Little is known about endocytosis, pH-dependence, and uncoating of infectious HNoV; however, studies of both FCV and MNoV can provide valuable insight. FCV entry is both clathrin-dependent and pH-dependent, as entry requires the acidic environment of the endosome for uncoating [89,90]. In contrast, MNoV entry is cholesterol- and dynamin-dependent but pH-independent [88,91]. Whether the differences in endocytic pathway utilization and pH-dependence between FCV and MNoV are virus-specific, host species-specific, or cell-type specific remains to be determined. Elucidating the mechanism of norovirus internalization may inform our understanding about norovirus entry, pathogenesis, and immune sensing.

Interestingly, non-enveloped viruses including hepatitis A virus, poliovirus, rotavirus, and noroviruses can be secreted non-lytically from cells inside extracellular membrane-bound vesicles [92,93,94]. These vesicles, likely derived from multivesicular bodies, can contain a wide range of viral particle numbers, which can vary across viral species. Both enveloped and non-enveloped, or naked, virions can exist for a given virus; these different viral forms can confer different physiologic properties. For instance, virions in vesicles are more environmentally stable, more virulent per particle, and resistant to host antibody neutralization [92,93,94,95]. The presence of a vesicle surrounding what are classically considered non-enveloped virions presents a challenge in understanding how non-enveloped viruses escape the vesicle and interact with their cellular receptors. This is because the viral capsid proteins which determine receptor utilization and tropism are cloaked by the lipid bilayer inside an extracellular vesicle. The differences between enveloped and naked virion entry has been best described for the hepatitis A virus in which enveloped and non-enveloped virions were recently demonstrated to enter cells by distinct intracellular trafficking routes [92,95]. Naked hepatitis A virus enters in the late endosome, while enveloped hepatitis A virus requires trafficking to the lysosome, where its envelope can be degraded prior to subsequent virus–receptor interactions [95]. Similarly, both MNoV and HNoV contain free and vesicle-bound forms [93]. Little is known about the properties or consequences of vesicle-cloaked HNoV. However, both enveloped and naked MNoV require CD300lf for entry [93]. Whether the molecular, spatial, and temporal interactions with CD300lf differ between enveloped and naked MNoV is unclear.

Endosomal escape and viral uncoating, the last step in viral entry, culminate with the release of the viral genome into the host cytoplasm. Viruses can utilize many mechanisms for endosomal escape. Enveloped viruses directly fuse with the plasma or endosomal membrane, releasing their genome into the intracellular environment, while non-enveloped viruses can release their viral genomes through membrane lysis or utilize membrane-piercing structures [96]. Our understanding of *Caliciviridae* endosomal escape and uncoating was limited until recent work by Bhella and colleagues identified a possible mechanism of FCV genome release [34,77]. Binding of FCV to its receptor fJAM-A induces a conformational change in the capsid protein VP1, leading to a rotation of the entire FCV P domain (Figure 2C). At a single three-fold axis, a small pore in the virus capsid shell is formed, allowing the minor structural protein VP2 to exit the virion interior. VP2 binds to both the P1 and the P2 domains of VP1. Twelve VP2 molecules assemble to form a large funnel-shaped portal-like structure on the outer surface of the viral capsid, arranged about the pore. The tip of the protruding VP2 protein is hydrophobic, which may enable endosomal membrane penetration (Figure 1). Consistent with the known entry biology of FCV, this process is dependent on receptor binding and acidic pH [34,42,77,89]. The VP2 of noroviruses is known to be essential for infection, although it is dispensable for virion assembly [20,34,97,98,99]. Whether the VP2 of noroviruses undergoes a similar conformation remains an important future direction.

## 6. Concluding Remarks

Viral entry is the first and often rate-limiting step in viral infection and a critical determinant of host range, cell tropism, and pathogenesis. Our understanding about norovirus entry has been enhanced by a number of recent observations including the role of HBGAs, bile salts, and ceramide in viral attachment, the discovery of CD300lf as a receptor for MNoV, and the potential role of VP2 in viral genome release [25,26,27,31,32,33,34,38,71]. A better understanding of HNoV entry may lead to improved cell culture systems, novel therapeutic and vaccine strategies, and increased understanding on how HNoV establishes infection, evades the immune response, and causes disease. While the receptor and molecular mechanisms of HNoV entry remain uncharacterized, recent advances in HNoV cell culture systems and basic discoveries made in understanding MNoV and FCV entry are poised to reveal novel and critical aspects of HNoV biology.
